# Overcoming Solubility Challenges: Micronization of Berberine Using the GAS Antisolvent Technique

**DOI:** 10.1021/acsomega.5c01675

**Published:** 2025-10-22

**Authors:** Guilherme S. B. Sakata, Patricia Viera de Oliveira, Gean Pablo S. Aguiar, J. Vladimir Oliveira, Marcelo Lanza

**Affiliations:** † Department of Chemical and Food Engineering, 28117 Federal University of Santa Catarina, Florianópolis, SC 88040-900, Brazil; ‡ Environmental Sciences Division, Unochapecó, Chapecó, SC PO Box 1141, Brazil

## Abstract

Berberine is a compound widely used in Chinese herbal medicine. It can potentially treat diabetes, cholesterol, and mental illnesses and has antimicrobial effects. However, its application is limited due to its low solubility in water, thus limiting its bioavailability. Micronization techniques are used to increase the surface area and improve the dissolution rate. The micronized particles were produced using the antisolvent GAS technique at a temperature of 35 °C and 80 bar. A solubility study with acetone, dichloromethane, ethanol, methanol, 1-butanol, and 1-propanol was carried out. Fourier-transform infrared spectroscopy analysis confirmed the preservation of functional groups. Additionally, a reduction in particle size to 6.34 μm was achieved, which contributed to an increase of up to 18% in cumulative dissolution. Furthermore, an increase in melting temperature and dissolution rate was observed. Thus, the antisolvent GAS technique proves to be an efficient approach for particle micronization and the consequent enhancement of dissolution properties.

## Graphical abstract

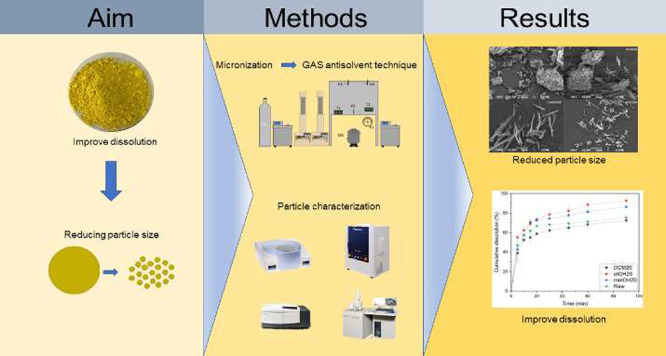

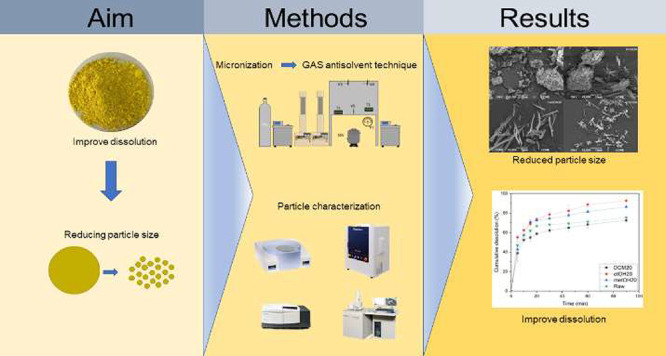

## 1 Introduction

Berberine is a natural alkaloid with a long history of use in traditional Chinese medicine. It exhibits a range of therapeutic potential and antimicrobial, antiprotozoal, and antidiarrheal properties.^1^ Beyond its medicinal properties, berberine is also used as a dye for coloring wood, wool, leather, and cotton.^2^ The compound’s pharmacological effects make it an attractive candidate for treating various diseases. For instance, it has shown promise in treating cancer, diabetes, depression, cardiovascular problems, and hypertension.^3^ Due to its versatile therapeutic potential, berberine has attracted significant attention from the scientific community and represents a promising avenue for drug development.

Due to its quaternary amine structure, berberine’s low solubility in water limits its effectiveness by reducing bioavailability and absorption in the gastrointestinal tract, despite its potential applications. Solubility plays a fundamental role in the oral administration of drugs.^4^ Compounds with low solubility often exhibit limited bioavailability,^5^ which can be overcome by reducing the particle size to increase the dissolution rate.^6^

Particle size reduction is one of the oldest techniques to improve compound solubility, as it increases the surface area available for solvation.^4^ Micronization techniques employ hydrodynamic and mechanical approaches to disrupt internal bonds, resulting in material disintegration, while the reduction of particle size to the micron scale induces alterations in structural, physicochemical, and functional properties.^7^

Traditional techniques encompass milling, high-pressure homogenization and spray drying.^8^ Nevertheless, these techniques exhibit several limitations that may impact the quality and efficacy of the final pharmaceutical product, including a broader particle size distribution (PSD) and the risk of thermal and chemical degradation, which present significant challenges to ensuring product stability and performance.^9^

To overcome the limitations of traditional techniques for particle size reduction, nonconventional techniques have been investigated as alternatives. One such promising technique is supercritical fluid techniques, which offer solvent-free products, greater diffusivity, and smaller particle sizes.^10^ Supercritical fluids are obtained by subjecting pure components to temperatures and pressures above their critical values, leading to the properties of both liquids and gases.^11^ For instance, carbon dioxide (CO_2_) has critical temperature and pressure values suitable for the precipitation of thermolabile materials without degradation, making it an environmentally friendly, nonflammable, and nontoxic option.^12^

Supercritical antisolvent techniques involve adding a second fluid to a mixture, which reduces the solubility of the solute in the solvent. Precipitation of the particles is controlled by diffusion of the antisolvent in the mixture, causing expansion of the volume and a reduction of solubility due to the drop in density.^13^ In the GAS (Gas Anti-Solvent) technique, the solute is initially dissolved in an appropriate organic solvent and transferred to a high-pressure vessel. Subsequently, supercritical carbon dioxide is introduced into the system, leading to volumetric expansion due to CO_2_ dissolution in the solvent. This phenomenon reduces the solubility of the solute, promoting its precipitation in the form of solid particles. The resulting particles, characterized by a modified size distribution, are recovered after the removal of excess solvent by the continuous flow of supercritical carbon dioxide.^14^ This technique offers versatility in solvent choice and has advantages, including using a large amount of solvent at once, achieving a narrow particle size distribution, and producing a solvent-free product.^15^

This study aims to investigate the potential use of supercritical techniques to increase the solubility and bioavailability of the compound by reducing its particle size. This approach may offer a promising avenue for enhancing the compound’s efficacy and expanding its potential applications by overcoming the limitations associated with low solubility.

## 2 Materials and Methods

### 2.1 Materials

Berberine chloride was purchased from Active Pharmaceutica (Florianópolis, Brazil), dichloromethane was purchased from Dinâmica Química Contemporânea (Indaiatuba, Brazil), ethanol (99.5%) was purchased from Êxodo Científica (Sumaré, Brazil), methanol was purchased from Êxodo Científica (Sumaré, Brazil), and carbon dioxide (99.9% in liquid phase) was provided by White Martins S/A (São Paulo, Brazil).

### 2.2 Parameter Optimization

Determination of the best conditions for the performance of the GAS antisolvent method is necessary to study the behavior of the phases of the system. The dissolution of a compressed fluid as an antisolvent causes supersaturation of the solution with consequent precipitation of the solute in the system, so it is consistent with concluding that the process of expansion of the liquid phase can represent the variation of the solute concentration in the liquid phase.

The precipitation of solids in the context of the GAS process is regulated by the phase equilibrium and kinetic. Profound insight into the phase equilibrium empowers the choice of solvents, temperatures, and pressures before the experiments. Employing a thermodynamic model facilitates the investigation into the expansion of volume within the system, consequently permitting choose the best variables.

The approach proposed by Mukhopadhyay and Dalvi (2004)^16^ was used, who suggested that the solute fraction in the ternary system is proportional to the partial molar volume fraction of the solvent (PVMF), eq 1:

$$x_{p}=\frac{\mathrm{FVPM}(T,P,X_{s})}{\mathrm{FVPM}(T,P^{o},X_{\mathrm{SO}})}x_{p}^{o}$$ 1

As the approach ignores the solute in the calculations, the partial molar volume can be obtained directly from the equations of state from the binary equilibrium data (CO_2_ + solvent). In this work, the Peng–Robinson equation, eq 2, was used with the van der Waals mixing rule, eqs 3 and 4, and the combination rule, eq 5.

$$P=\frac{RT}{v-b}-\frac{\alpha \left(T\right)}{v^{2}+2bv-b^{2}}$$ 2

$$\alpha =\underset{i}{\sum }\underset{j}{\sum }x_{i}x_{j}a_{ij}$$ 3

$$b=\underset{i}{\sum }x_{i}b_{i}$$ 4

$$a_{ij}=\sqrt{\left(\alpha_{i}\alpha_{j}\right)}\left(1-k_{ij}\right)$$ 5

The partial molar volume of the solvent in systems with CO_2_ was calculated from binary system data (CO_2_ + solvent) taken from the literature and presented in Table 1.

**Table 1:** Binary Phase Equilibrium Data

**solvent**	***k**_**ij**_*	***l**_**ij**_*	**reference**
acetone	0.0196	0.0241	^17^
dichloromethane	0.0673	–0.0173	^18^
ethanol	0.0865	–0.1320	^19^
methanol	0.0660	0.0180	^20^

Considering that the driving force for the precipitation of the particles is the change in the partial molar volume, which coincides with the decrease in the partial molar volume with the solute concentration in the liquid phase,^21^ the change in the partial molar volume was calculated according to eq 6.

$$\overset{®}{v_{2}}=\left\lfloor v-x_{1}{\left(\frac{\partial v}{\partial x_{1}}\right)}_{P,T}\right\rfloor$$ 6

Eq 7 represents the concentration of the solute in the liquid phase of the system:

$$x_{3}(T,P)=\frac{\frac{\left(1-X_{1})\overset{®}{v_{2}}(T,P,X_{1}\right)}{v(P,X_{1})}}{\frac{\left(1-X_{10})\overset{®}{v_{2}}(T,P_{0},X_{10}\right)}{v(P_{0},X_{10})}}x_{30}(T,P_{0})$$ 7

where *x*_3_ = *X*_3_(1 – *x*_1_), *x*_1_ = *X*_1_ (1 – *x*_3_), and *x*_1_+ *x*_2_ + *x*_3_ = 1, with x_30_ the mole fraction of solute in the ternary system and *X*_10_ the fraction of CO_2_ in the solvent on a solute-free basis at the reference pressure *P*_0_ and *X*_1_ the fraction of CO_2_ on a solute-free basis.

### 2.3 Gas Antisolvent Procedure

Berberine was micronized using the antisolvent gas technique based on the studies of Pessoa et al. (2019)^22^ and Sakata et al. (2021).^23^ First, the solution was prepared at 1 mg/mL with the selected solvents and then added to the cell. The CO_2_ was introduced into the cell at a flow rate of 10 and 20 mL/min until the pressure reached 80 bar, with an operating temperature of 35 °C. The mixture was held at 80 bar for 10 min for homogenization. The solvent was removed by passing CO_2_ at constant pressure with open inlet and outlet valves. Once all the solvent had been removed, the reaction cell was depressurized, and the particles were collected.

### 2.4 Thermal Analysis

The thermal properties of the compound have been analyzed utilizing differential scanning calorimetry analysis (Jade-DSC PerkinElmer). Raw and processed Berberine were investigated over a temperature range of 40 to 300 °C at a heating rate of 10 °C/min and a nitrogen flow rate of 20 mL/min.

### 2.5 Fourier Transform Infrared Spectroscopy (FTIR)

FTIR analysis (Agilent Technologies – Cary 660) investigated possible changes in the molecule’s functional groups. The analysis was performed at a wavelength of 4000 to 400 cm^–1^ with a resolution of 4 cm^–1^, and 32 scans and KBr pellet were used as a background reference spectrum.

### 2.6 Scanning Electron Microscopy (SEM)

Morphology and particle size were examined by scanning electron microscopy (JEOL JSM-6390LV.). Length and width measurements of 200 particles were obtained for each sample using Size Meter Software (version 1.1).

### 2.7 Crystalline Structure

X-ray diffraction (XRD) analysis was used to observe the crystalline structure of the particles produced compared to the untreated sample. Diffraction data were collected between 2 and 40° 2θ degrees with a step size of 0.02°/s using a MiniFlex 600 XRD Rigaku.

### 2.8 Dissolution Study

The dissolution investigation was conducted by drawing upon the research of Cheng et al. (2016)^24^ and Oliveira et al. (2023).^25^ A 10 mg sample was introduced into 100 mL of distilled water, which was then subjected to continuous agitation at a temperature of 37 °C and a speed of 100 rpm. To maintain a constant volume, 1 mL aliquots of the solution were promptly extracted and replaced. Subsequently, the samples were subjected to filtration utilizing a 0.45 μm PTFE filter, and the concentration of berberine was quantified using a UV–vis spectrophotometer set at a wavelength of 342 nm.

### 2.9 Solubility Analysis

The solubility study of the compound in different solvents was carried out. Acetone, ethanol, methanol, 1-propanol, 1-butanol, and dichloromethane were used. An excess compound was added to 100 mL of solvent at 25 and 35 °C. The solution was agitated at 100 rpm for 24 h to achieve solid–liquid equilibrium. It was then left for 2 h without stirring to allow the sedimentation of the insolubilized particles. An aliquot was taken and analyzed with a spectrophotometer.

## 3 Results and Discussion

### 3.1 Solubility

The solubility values for berberine in the solvents and temperatures studied are shown in Table 2. It is seen that the solubility of berberine increases with increasing temperature, this indicates that the dissolution of berberine in all solvents has an enthalpy greater than zero, whereas according to Petrucci et al. (2017),^26^ systems that have Δ*H*_sol_ > 0 on heating increase the solubility in the solvent. Methanol has the highest solubility, reaching 35 mg/L at 35 °C, followed by 1-butanol, 1-propanol, ethanol, dichloromethane (DCM), and acetone.

**Table 2:** Solubility of Berberine in Various Solvents at 25 and 35 °C

	solubility (mg/L)	
solvent	25 °C	35 °C
acetone	0.16 ± 0.004	0.20 ± 0.007
dichloromethane	1.27 ± 0.032	3.47 ± 0.091
ethanol	2.82 ± 0.086	4.57 ± 0.254
methanol	15.37 ± 1.614	35.00 ± 1.98
1-propanol	3.00 ± 0.168	4.33 ± 0.082
1-butanol	5.40 ± 0.578	9,49 ± 0.282

### 3.2 Parameter’s Optimization

Based on the concepts previously discussed in the literature, optimal conditions for realizing the antisolvent GAS are related to the partial molar volume of the solvent in the binary system.^21,27,28^ Calculations were performed to select the best pressure and temperature conditions, assuming the solvent-antisolvent interaction is more significant than the solute–solvent-antisolvent interaction.^29^

Following the approach proposed by Mukhopadhyay (2003),^29^ the molar partial volume reduction of the solvent was calculated using the equations presented in the materials and methods. The data for the binary systems were taken from literature sources shown in Table 1.

The behavior of the partial molar volume variation as a function of the variation of the mole fraction of the antisolvent is shown in the following Figure 1.

**Figure 1:**
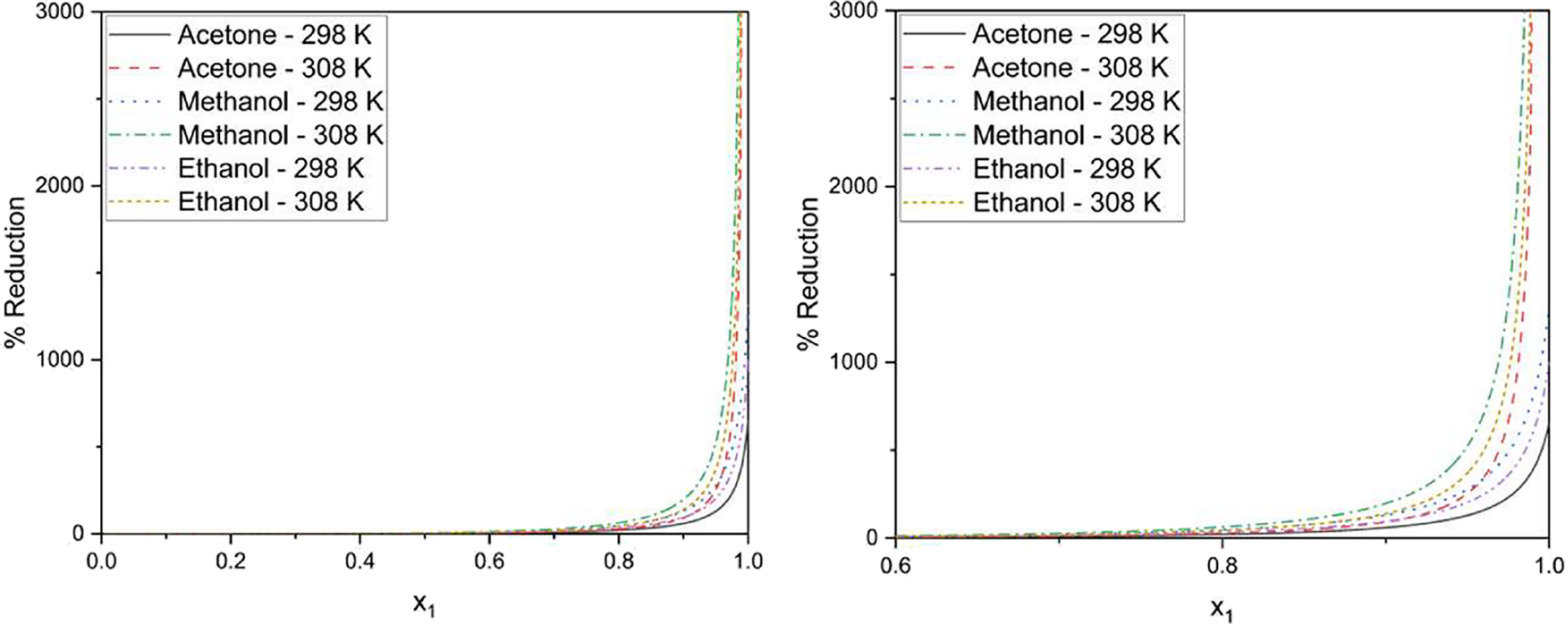
Relative molar volume reduction in binary system carbon dioxide (1) + solvent (2).

It is possible to observe that for each temperature, a profile in the volume variation behavior is obtained. Compared to the profile presented for the temperature of 298 K, the behavior presented for the temperature of 308 K shows a more accentuated volume reduction for high concentrations of antisolvent. Micronization processes require rapid crystallization, as the approach used relates the solute fraction to the partial molar volume of the solvent. The behavior presented for the highest temperature in all solvents proves to be the most suitable for the process since it shows a more pronounced reduction when compared to the lowest temperature.^29^

The operating pressure was obtained by graphing the molar volume variation versus pressure, as shown in Figure 2, and a minimum point could be observed from which a sharp increase in volume expansion occurred.^28^ The calculation of the partial molar volume expansion was obtained using eq 6. De La Fuente Badilla et al. (2000)^21^ suggest in their studies that the minimum molar volume reduction pressure may be the optimum pressure for the GAS process.

**Figure 2:**
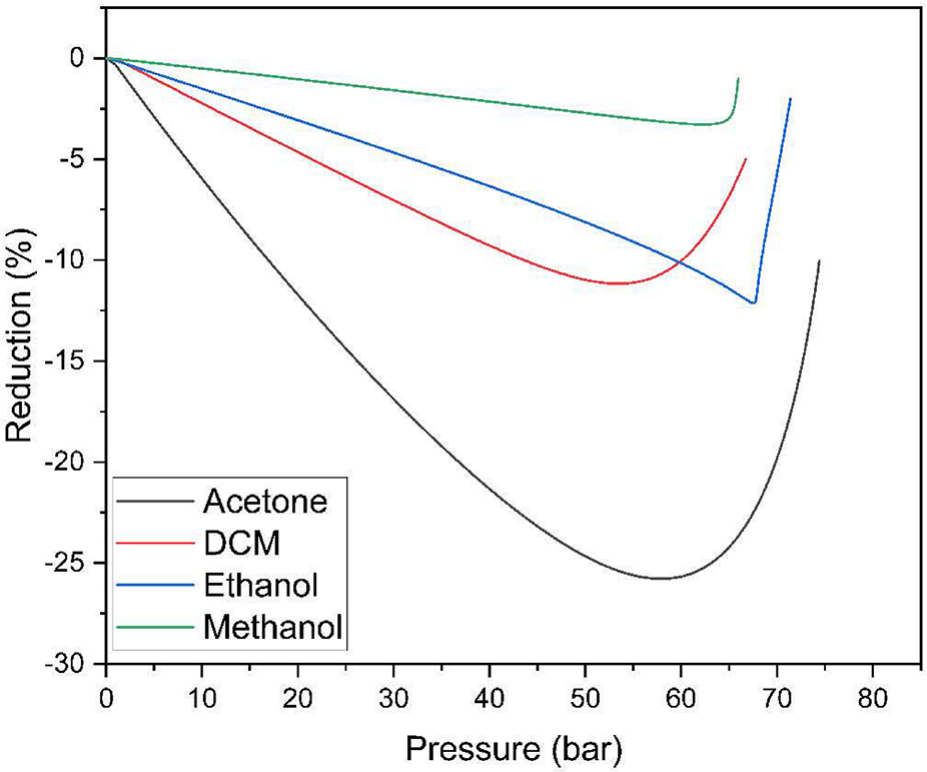
Relative expansion of liquid phase for binary system carbon dioxide (1) + solvent (2).

Figure 3 shows that different solvents give different composition profiles as a function of pressure for a given temperature. These profiles are an important source of information about the particle size result.

**Figure 3:**
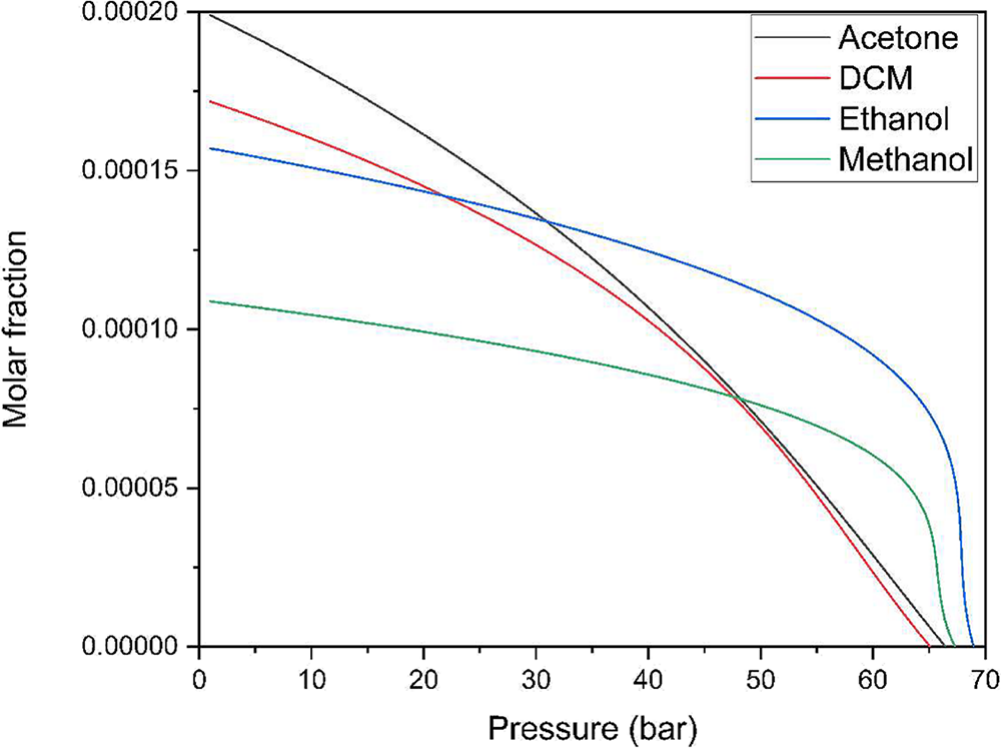
Predicted solubility of berberine in a solvent at 308 K.

Systems in which the solubility of the solid decreases slowly with the addition of CO_2_ tend to have larger particle sizes due to continuous and relatively slow precipitation. In contrast, systems, where the drop in solubility occurs more rapidly tend to have smaller and narrower particle sizes due to rapid and uniform precipitation in this range.^30^ Based on the solute concentration profiles observed across distinct solvent mediums, as depicted in Figure 3, it can be deduced that particles generated through the utilization of dichloromethane (DCM) or acetone exhibit a relatively greater mean particle size in comparison to those synthesized within ethanol or methanol environments.

This approach has been applied to crystallization process optimization, CO_2_ solubility prediction, particle formation optimization, and coprecipitation in supercritical processes.^31–35^

### 3.3 Differential Scanning Calorimetry (DSC)

Berberine has its point of transition from the solid phase to the liquid phase at a temperature of 189 °C^36,37^ and Δ*H*_fusion_ = 112,35 J/g.^38^ An endothermic peak between 95 and 130 °C due to dehydration of berberine.^39,40^ The DSC curves for the samples studied are shown in Figure 4.

**Figure 4:**
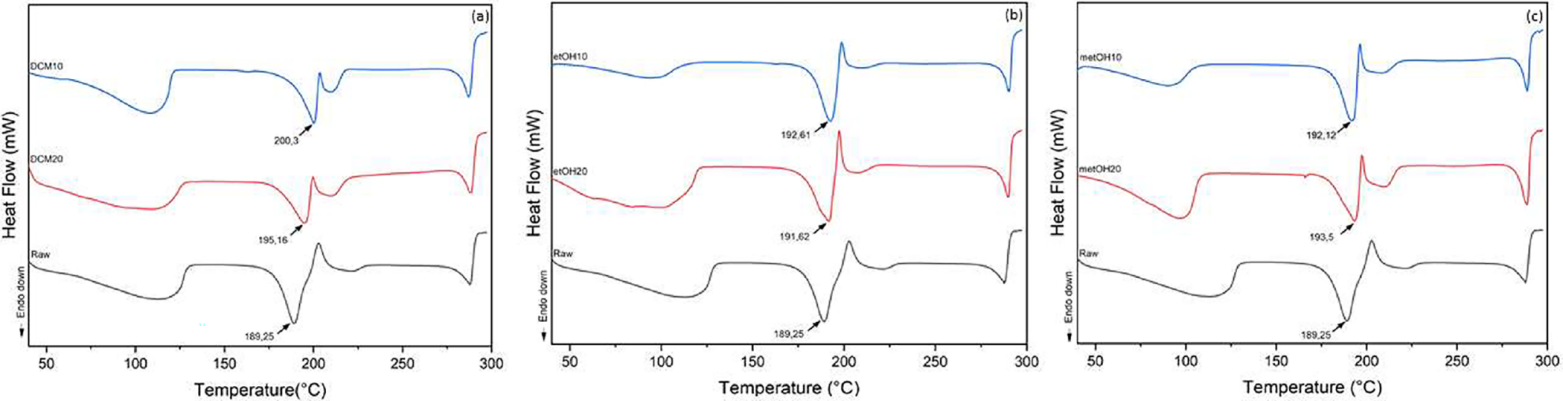
Differential scanning calorimetry of raw material and micronized berberine using (a) dichloromethane, (b) ethanol, and (c) methanol in different antisolvent flow rates with 10 and 20 mL/min.

It is possible to observe changes in the thermal properties of the compound, a reduction in the Δ*H*_fusion_ of the samples accompanied by an increase in the melting temperature. These changes can be explained by changes in the crystalline structure of the compounds, which can modify properties such as melting temperature, dissolution rate, and solubility, among others.^41^

However, the melting temperature values obtained from the samples, which ranged from 191.62 to 200.3 °C, have already been reported in the literature and presented as the melting point of the compound, as Kohli et al. (2021)^42^ reported a melting point with a peak at 191.58 °C, and Feng et al. (2018),^43^ who reported a peak at 201.9 °C, among other studies that reported intermediate values.^44–47^

Another point that can be observed in the DSC plot is the presence, after the melting peak, of an exothermic event followed by an endothermic event; this sequence of events is characteristic of compounds that have a characteristic polymorphism of the enantiotropic type, in which one crystalline form melts at a lower temperature and instantly crystallizes into a second polymorphic form that melts at a higher temperature.^48^

### 3.4 Fourier Transform Infrared Spectroscopy (FTIR)

The spectra of the particles obtained with each solvent (DCM, ethanol, and methanol) were obtained and are shown in Figure 5, separated by the solvents used in the preparation of the particles in comparison with the commercial compound.

**Figure 5:**
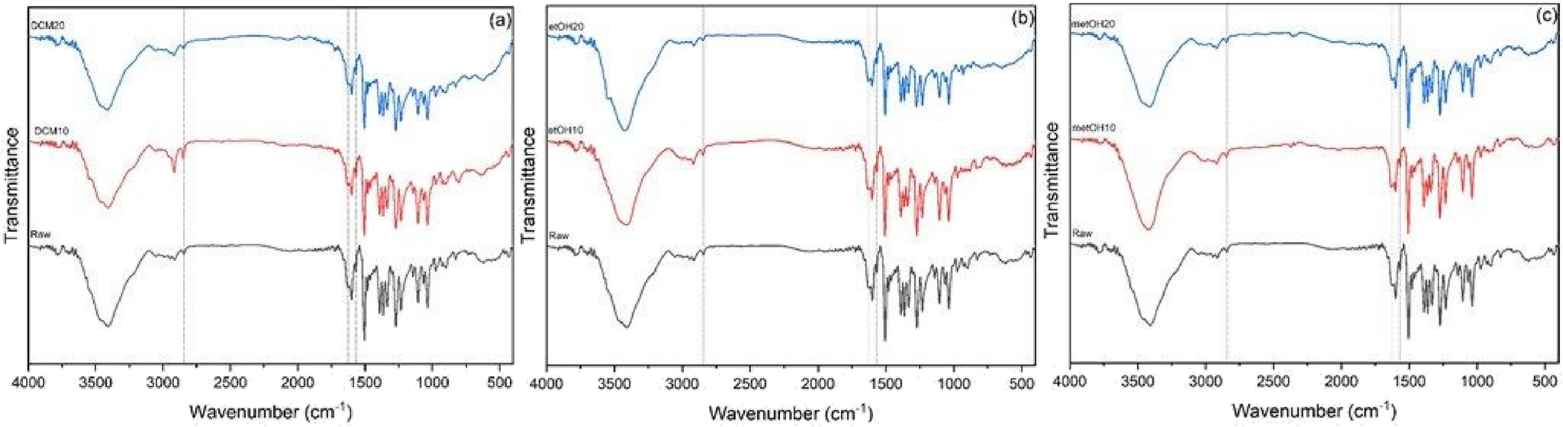
FTIR spectrum of raw material and micronized berberine using (a) dichloromethane, (b) ethanol, and (c) methanol in different antisolvent flow rates with 10 and 20 mL/min.

Pure berberine has characteristic peaks present at 2844 cm^–1^ corresponding to the methoxyl group (R–O–CH3),^49^ 1635 cm^–1^ the iminium cation (C=N+),^50^ at 1569 cm^–1^ the stretching gives the aromatic C=C bond,^51^ these peaks were also observed in the samples prepared. In addition to the characteristic peaks, there are also peaks between 1100 and 1272 cm^–1^ related to C–O and C–N stretches^52^ and 1035 cm^–1^ related to the C–H vibration.^53^ The spectra show that the characteristic peaks of the pure sample are present in all the processed particles, indicating no structural changes in the processed particles.

### 3.5 Crystalline Structure

Berberine, in both raw and micronized forms, was analyzed by powder X-ray diffraction to assess potential modifications in the crystalline structure of the compounds. The corresponding results are shown in Figure 6.

**Figure 6:**
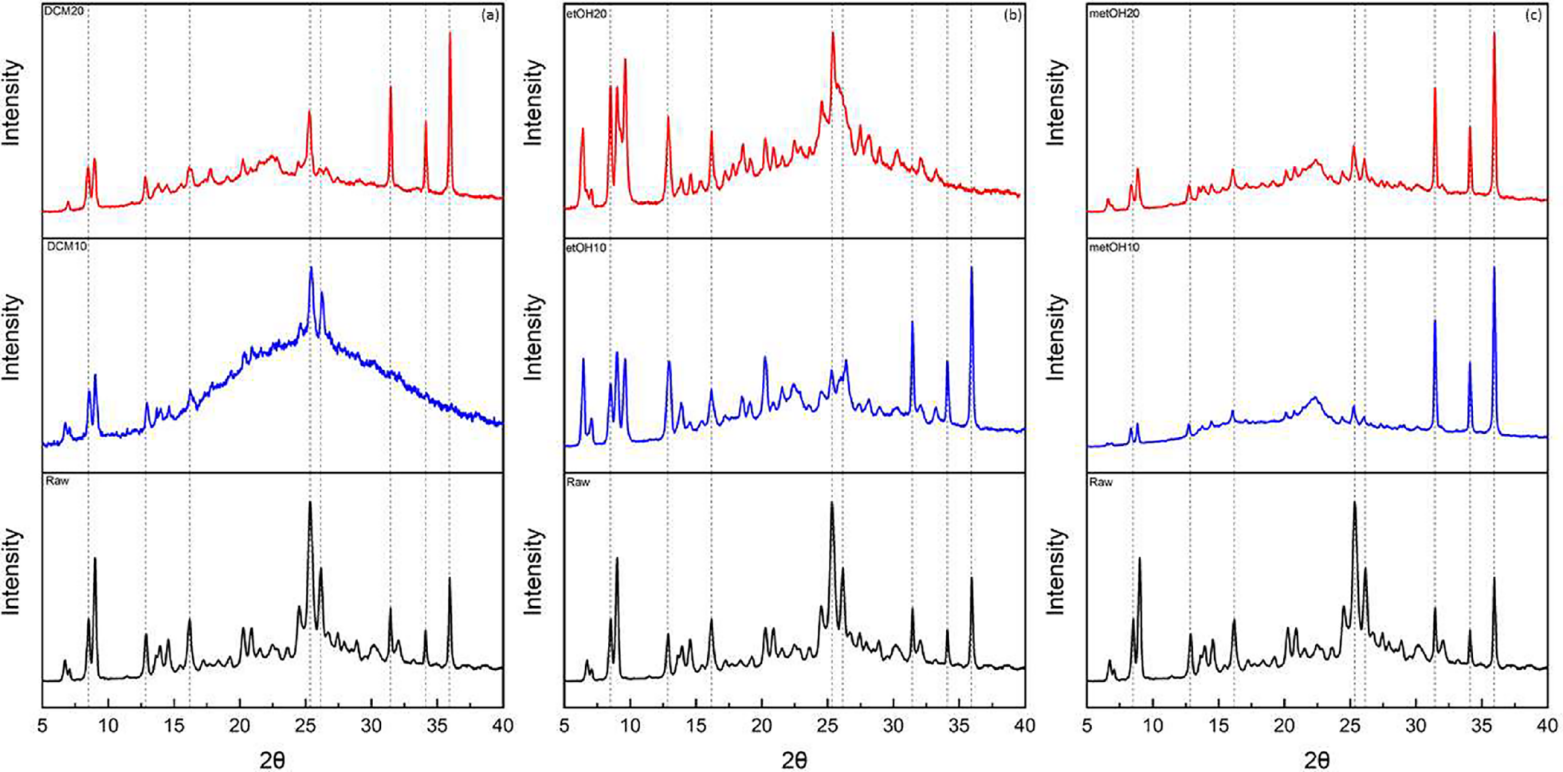
X-ray diffraction of raw and micronized berberine using (a) dichloromethane, (b) ethanol, and (c) methanol in different antisolvent flow rates with 10 and 20 mL/min.

Berberine shows characteristic crystallinity peaks for 2θ values of 8.5°, 12.86°, 16.2°, 25.35, and 26.16° as previously reported in the literature.^45,54^ Characteristic peaks were also observed at 31.44°, 34.12°, and 35.92° in the pure compound.

For the sample with DCM, due to the presence of the elevation halo in the diffractogram, more present in the 10 mL/min samples, a particle with an amorphous structure was obtained. In the 20 mL/min sample, the crystalline peaks are preserved, but their intensity is reduced, whereas in the 10 mL/min sample, only the characteristic peaks below 10°, the peaks at 25.35° and 26.16°, are preserved.

The ethanol particles show peak shifts in both CO_2_ flow rates, with a peak shift from 6.74° to 6.44° and a new crystalline plane formed at 9.6° at 10 mL/min. At the 20 mL/min flow rate, there was also a shift and appearance of new peaks. This was the case for the change from 6.74° to 6.50°, at 25.35° to 25.50°, and the appearance of a new peak at 9.71°.

The sample with methanol at 10 and 20 mL/min showed the same characteristics, with peaks shifting from 8.5°, 9°, and 16.2° to 8.3°, 8.8°, and 16.04°, respectively, and the 32.03° peak disappearing.

Changes in the intensity of reflections and shift of reflections from pure compound to samples may indicate changes in the crystalline form of the compound, which may confer new functionalities.^55^ The relative intensity of the peaks can be analyzed due to the crystal size. Particles with reduced intensity may result from reduced crystallinity or particle size.^6^ The appearance of new reflection peaks or disappearing reflections can indicate the formation of a new polymorphic form of the solid.^56^

The diffractograms of these samples were compared with those of previously published polymorphs to observe the differences in the crystal structure of the particles produced with ethanol. The Cambridge Structural Database (CSD)^57^ provides various crystallographic data for berberine, as shown in Table 3. These data were compared with the diffractograms of the ethanol-produced particles shown in Figure 7.

**Table 3:** Berberine Crystallographic Data

**compound**	**database identifier**	**reference**
berberine chloride dihydrate	XUNFES	^58^
berberine chloride dihydrate	XUNFES01	^59^
berberine chloride dihydrate	XUNFES02	^60^
berberine chloride tetrahydrate	YUJHAM	^61^
berberine chloride tetrahydrate	YUJHAM01	^59^
berberine chloride ethanol solvate hemihydrate	YUJHIU	^61^
berberine chloride methanol solvate	XEBCUG	^62^

**Figure 7:**
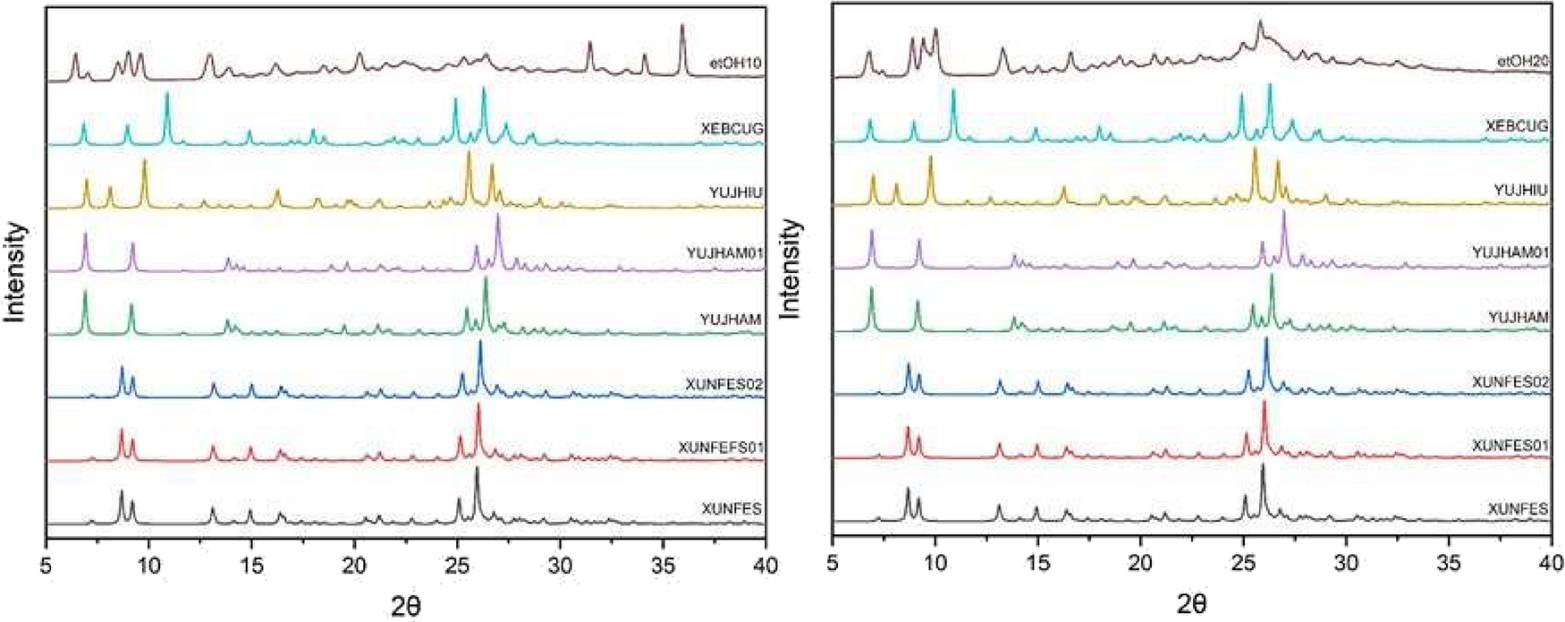
X-ray diffraction of micronized berberine in ethanol and published data for berberine, berberine chloride dihydrate (XUNFES), berberine chloride dihydrate (XUNFES01), berberine chloride dihydrate (XUNFES02), berberine chloride tetrahydrate (YUJHAM), berberine chloride tetrahydrate (YUJHAM01), berberine chloride ethanol solvate hemihydrate (YUJHIU), and berberine chloride methanol solvate (XEBCUG).

It can be observed that there was no peak coherence between the samples (etOH10 and etOH20) and previously published polymorphs, indicating that the crystalline planes present in the sample were not yet cataloged. However, determining the occurrence of a new polymorphic form would require an in-depth investigation employing techniques such as Solid-State Nuclear Magnetic Resonance (SSNMR), Single-Crystal X-ray Diffraction (SC-XRD), and thermal microscopy analyses. However, such an approach was beyond the scope of the present study.

### 3.6 Particle Size

The particle size and morphology obtained before and after the GAS antisolvent process were scrutinized via scanning electron microscopy (SEM), as depicted in Figure 8. This analysis facilitated particle size assessment by considering the maximum number of particles measurable within the acquired images.

**Figure 8:**
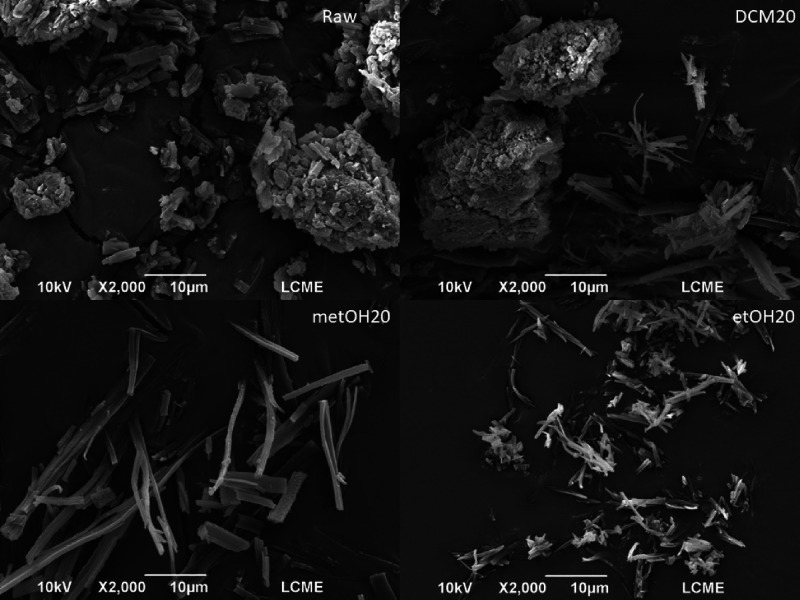
SEM of raw and micronized berberine produced in dichloromethane, ethanol, and methanol using 20 mL/min as the antisolvent flow rate.

The raw BRB exhibits particles with irregular morphology and a range of sizes, and the DCM-formed particles exhibit the same traits with various shapes and sizes. The particles produced by ethanol and methanol have similar morphologies and needle-like features, with a narrow width and a greater length measurement. As seen in Figure 3, where the reduction in the compound’s solubility in DCM is much less pronounced, these differences can be explained by the likely differences in the precipitation of the particles. This allows for the possibility of particle agglomeration.

Similar patterns can be seen in the compound’s solubility in ethanol and methanol, where a sharp decline in solubility leads to rapid precipitation of the particles. This similar profile may explain the similar morphologies between the solvents. Particles made with ethanol and methanol were anticipated to be smaller than those made with DCM, as demonstrated in the study of the molar partial volume reduction approach. Due to the samples’ needle-like appearance, length and width measurements were taken for ethanol and methanol.

The measured values of the produced particulates and raw samples are shown in Table 4. Compared to commercial particles, the micronized particles’ average particle size is reduced in length and width.

**Table 4:** Particle Size of Raw and Micronized Berberine Using Dichloromethane, Ethanol, and Methanol in Different Antisolvent Flow Rates with 10 and 20 mL/min

sample	length (μm)	width (μm)
DCM10	16.01 ± 8.779	9.05 ± 5.24
DCM20	13.20 ± 10.70	5.62 ± 5.10
etOH10	13.31 ± 7.69	1.75 ± 0.74
etOH20	9.88 ± 6.06	1.51 ± 0.71
metOH10	11.70 ± 7.83	1.33 ± 0.66
metOH20	6.34 ± 3.87	1.03 ± 0.46
Raw	13.24 ± 8.66	6.39 ± 5.39

Nonmicronized berberine particles exhibit average length and width values of 13.24 and 6.39 μm, respectively, under standard conditions. However, the optimal condition yields dimensions of 6.34 and 1.03 μm for length and width, respectively. Notably, a decrease in both sizes is evident for samples experiencing a higher CO_2_ leakage. This reduction can be attributed to the rapid decline in mole fraction resulting from an increased flow rate, which limits the available time for particle agglomeration.^31^

### 3.7 Dissolution

The principal aim of micronization is to enhance solubility and subsequently improve bioavailability. The smallest sample sizes were analyzed for each solvent to investigate the cumulative dissolution. Specifically, this study generated the samples using a CO_2_ flow rate of 20 mL/min due to the smaller particle size obtained by this condition. The dissolution profile is shown in Figure 9.

**Figure 9:**
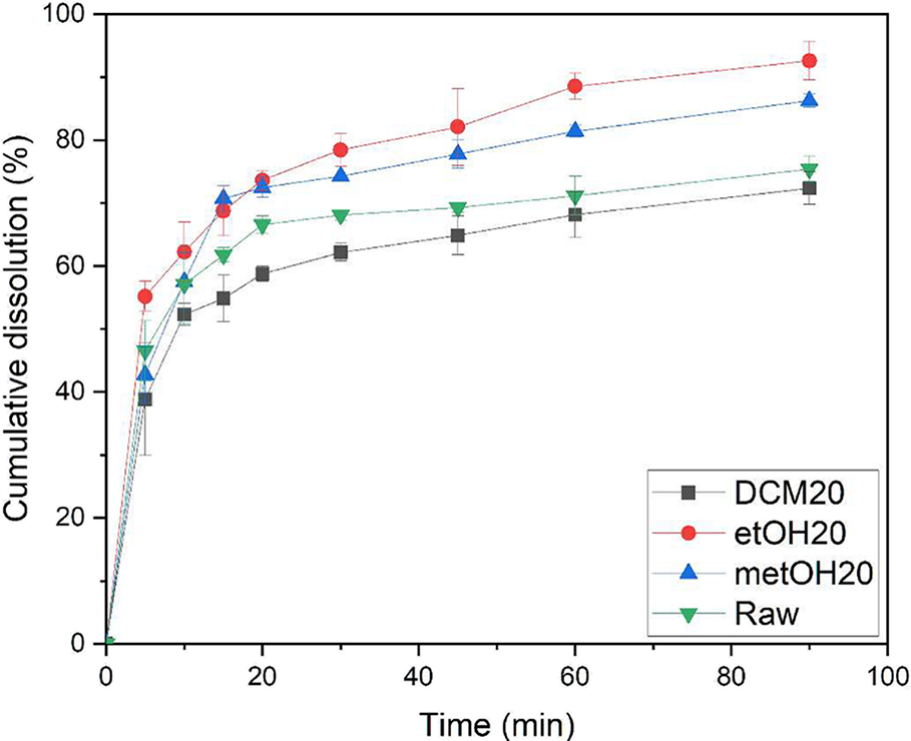
Dissolution rate of raw and micronized berberine produced in dichloromethane, ethanol, and methanol using 20 mL/min as antisolvent flow rate.

The graph depicts the temporal evolution of dissolution. It is evident that the samples exhibiting the smallest particle size, as indicated by the Noyes-Whitney equation, demonstrate the highest dissolution rates.^63^ Furthermore, micronized luteolin particles, produced using the same technique, exhibit a notable enhancement in cumulative dissolution, affirming the efficacy of the micronization method in augmenting dissolution rates.^64^

Specifically, when micronized with ethanol, the samples exhibit an accumulated dissolution approximately 18% higher than the commercial sample at the end of the 90 min time frame. Dissolution rate enhancements comparable to those observed in the present study have been reported for micronized particles of allopurinol,^65^ imiquimod,^66^ and fenofibrate.^67^ The modest enhancement in the dissolution rate of the compound can be attributed to the propensity of the particles to form agglomerates and retain adsorbed air, thereby hindering their dispersion in aqueous media.^68^

## 4 Conclusions

Micronization was accomplished utilizing the antisolvent technique, yielding desirable results. Significant observations were made through an examination of the partial molar volume, the relationship between solute fraction and pressure variation, the influence of temperature on volume variation, and the distinct profiles of each solvent. Solvents exhibiting a gradual decrease in fraction were found to produce larger particles. Micronized particles exhibited an enhanced dissolution rate compared to their commercial counterparts. Furthermore, the functional groups of the micronized particles remained intact. Alterations in melting temperature and enthalpy were observed, aligning with documented values in the existing literature, thereby indicating the presence of enantiotropic polymorphism. DCM yielded an amorphous structure, while ethanol displayed novel crystalline peaks.
